# A semantic rule based digital fraud detection

**DOI:** 10.7717/peerj-cs.649

**Published:** 2021-08-03

**Authors:** Mansoor Ahmed, Kainat Ansar, Cal B. Muckley, Abid Khan, Adeel Anjum, Muhammad Talha

**Affiliations:** 1 Department of Computer Science, COMSATS University Islamabad, Islamabad, Pakistan; 2 Innovation Value Institute, Maynooth University, Maynooth, Ireland; 3 UCD College of Business and Geary Institute, Dublin, Ireland; 4 Department of Computer Science, Aberystwyth University, Aberystwyth, UK

**Keywords:** Digital fraud, Semantic web, Knowledge base, Alert model, Database

## Abstract

Digital fraud has immensely affected ordinary consumers and the finance industry. Our dependence on internet banking has made digital fraud a substantial problem. Financial institutions across the globe are trying to improve their digital fraud detection and deterrence capabilities. Fraud detection is a reactive process, and it usually incurs a cost to save the system from an ongoing malicious activity. Fraud deterrence is the capability of a system to withstand any fraudulent attempts. Fraud deterrence is a challenging task and researchers across the globe are proposing new solutions to improve deterrence capabilities. In this work, we focus on the very important problem of fraud deterrence. Our proposed work uses an Intimation Rule Based (IRB) alert generation algorithm. These IRB alerts are classified based on severity levels. Our proposed solution uses a richer domain knowledge base and rule-based reasoning. In this work, we propose an ontology-based financial fraud detection and deterrence model.

## Introduction

Money laundering is the process of turning illegal currency into legal. Economies across the globe have taken strict actions to curb money laundering schemes. Various methods are being used to record and report suspicious financial activities. Customers’ financial behaviors are being monitored based on their transactional trends. Abnormal foreign and domestic transactions of sizeable amounts often point towards money laundering. Recently, researchers have proposed different approaches to resolve this problem (Dal et al., 2018). Despite the existence of anti-money laundering techniques, fraudulent entities often have their ways. For example, fraudsters often break the amount into smaller units to avoid suspicion. Various financial frauds have surfaced over the years. For example, credit card scams, fraudulent insurance claims, etc.

User’s behavioral, statistical, and social analyses are being done to detect financial frauds. Researchers have also analyzed abnormal financial activities using data mining. Abdallah, Maarof & Zainal (2016) have explored various fraud detection techniques in their research. Abdallah et al. focused on telecommunication, health care, and insurance frauds. A system is required to address the threat of financial fraud. A solution that could generate alerts on suspicious transactions is the need of the hour. To further this cause, we present an ontological fraud detection mechanism. The proposed model generates fraud alerts on suspicious transactions. It also tags each alert with a severity level as discussed in ‘System Model and Problem Formulation’.

### Ontologies *vs.* database models

Currently, ontologies are the best way to represent knowledge in a dynamic environment. It makes knowledge shareable and reusable. Additionally, ontologies can describe the terms and vocabularies of a domain. Ontologies allow knowledge bases and logic to be combined and turned into inferred knowledge via an inference engine. By using ontologies, we can reduce the modeling cost. One can extend and reuse ontologies for different applications and domains. The two basic data representational models are databases and ontologies. The relational databases have been in use for quite some time for storing and querying data. On the other side, ontologies with context have appeared as an alternative to databases with more enriched meaning. Ontologies make knowledge shareable and reusable (Dadjoo & Kheirkhah, 2015). The reasoning capabilities of ontologies make it possible to derive implicit facts from the knowledge base.

A database is usually designed for a specific application. For every application, one must create a new database. However, ontologies can be reused in different applications and domains as per need. Ontologies also help us in expressing the semantics in a better way as compared to databases. Since databases are schema-oriented, strict schema rules must be followed to create new records. The reasoning/inferring capability of ontologies makes it possible to produce new knowledge. Ontological classes, properties, and axioms can be mapped to a database’s tables, attributes, and constraints, respectively (Martinez-Cruz, Blanco & Vila, 2012).

### Motivation

A hybrid solution based on data mining and a complex network classification algorithm is presented by Zanin et al. (2018). The authors proposed a solution to detect credit card fraud. Our proposed solution has fraud detection and deterrence capabilities. Our work derives facts from the given knowledge base based on logical reasoning. These facts are not described explicitly and are referred to as inferred knowledge. Our solution also generates alerts on suspicious transactions along with their severity level.

### Our contribution

In this work, we have proposed an ontology-based alert model for Financial Fraud Detection and Deterrence (FFD). The main contributions of this research work are:

i. We have created a comprehensive FFD ontology with 40 classes and sub-classes. The FFD ontology identifies suspicious transactions based on customers’ bank transactions. It also relates transactions with each other to find out any malicious behavior.
ii. We have also developed rules using the Apache Jena framework, for our fraud alert systems.
iii. The proposed ontology-based alert model has extra features for money laundering detection and deterrence.
iv. The proposed IRB alert generation algorithm stops fraud before it occurs.
v. Our work also adds a taxonomy of literature review to facilitate a bird’s eye view.

The rest of the paper is structured as follows: ‘Related Work’ presents related work. In ‘Types of Fraud’, types of fraud are explored. Furthermore, an explanation of our proposed system is discussed in ‘System Model and Problem Formulation’. In addition, ontology construction methodology is presented in ‘Ontology Construction Methodology’. Moreover, formal representation of FFD ontology is discussed in the subsection of ‘Ontology Construction Methodology’. ‘FFD Ontology Implementation’ and ‘Ontology Validation’ present the ontology implementation and its validation, respectively. The evaluation setup, results, and discussion are presented in ‘Simulation Setup and Results’. Finally, ‘Conclusions and Future Work’ presents the conclusion.

## Related Work

**Figure 1:**
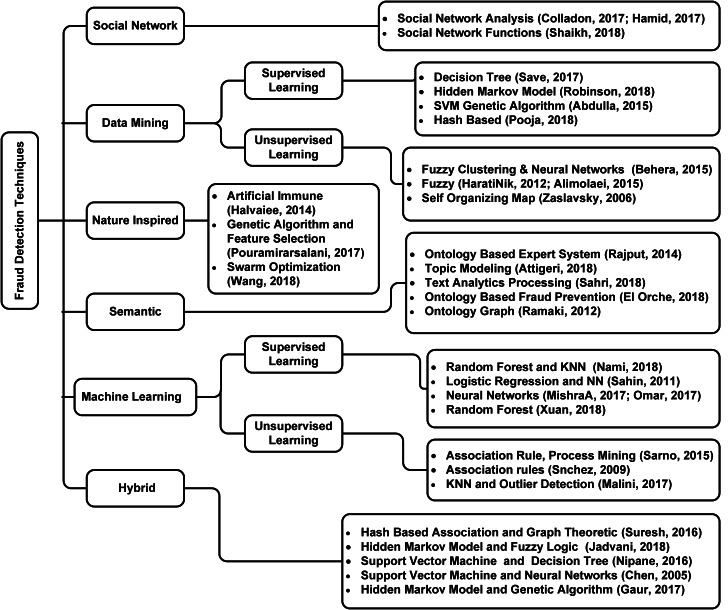
Taxonomy of literature review.

Technological advancements have come a long way. With technology being everywhere, the number of fraudulent activities has increased substantially. Researchers have analyzed a lot of fraud detection techniques over the years: Chen et al. (2005); Gaur et al. (2017); Jadvani et al. (2018); Nipane et al. (2016); Omar, Johari & Smith (2017); Pouramirarsalani, Khalilian & Nikravanshalmani (2017); Wang et al. (2018). Figure 1 shows the detailed taxonomy of fraud detection techniques reviewed in this work.

There are two aspects of digital fraud: prevention and detection. Prevention is the first wall of defense and usually allows systems to deter any threat. Detection is a means of identifying an ongoing or already occurred attack (Abdallah, Maarof & Zainal, 2016). Fraud prevention and fraud detection are two different aspects of a financial system. Prevention is the first layer, whereas detection is the next layer of protection to secure the system against fraud (Abdallah, Maarof & Zainal, 2016). The authors of West & Bhattacharya (2016) have explored detection techniques for many fraud types, *i.e.*, credit cards, financial statements, insurance, securities, and commodities frauds, etc.

Metadata provides basic public information about an object. Sen & Dash (2013) addressed the issue of misclassification and correct classification of fraudulent activities. The authors used meta-learning and various other classifier techniques in their research. In Delamaire, Abdou & Pointon (2009), the authors described various types of fraud *e.g.,* behavioral, application, bankruptcy, and theft frauds. Lata, Koushika & Hasan (2015) described the various financial practices to detect frauds. Frauds can be detected by supervised methods (classification) or unsupervised methods (behavior changes or unusual transactions). These types of financial practices are discussed by authors in the paper Lata, Koushika & Hasan (2015).

The use of different data mining techniques individually or in combination may return better results. Nami & Shajari (2018) proposed a two-stage method based on random forest and K-Nearest Neighbor (KNN) for payment card fraud detection. An algorithm based on reverse KNN (classification method) is proposed in Ganji & Mannem (2012) for credit card fraud detection.

Zaslavsky & Strizhak (2006) suggested the use of Self Organizing Maps (SOM) for developing fraud detection systems. By using SOM, changes in the behaviors of individuals can be detected. HaratiNik et al. (2012) proposed a fuzzy rule-based expert system for credit card fraud detection. The authors of (Alimolaei, 2015) developed a system for detecting users’ abnormal behavior on internet banking.

Data mining-based supervised learning methods were used by authors of Save et al. (2017). The authors developed a system for Credit Card (CC) fraud detection. The system was based on the decision tree method with the integration of the algorithm. The authors of Robinson & Aria (2018) used Hidden Markov Model to automatically detect prepaid card fraud. The proposed system was tested on a real transactional dataset. Several unsupervised learning techniques were used for detecting frauds in the financial sector (Makki et al., 2017).

Data mining can help in detecting fraudulent transactions. Patil & Lilhore (2018) discussed CC fraud detection by using machine learning and data mining. The authors’ solution used real transactional data of credit cards. The authors of (Zanin, 2018) proposed a hybrid of data mining and complex network classification algorithm. The solution proposed enabled the authors to detect CC fraud. Quah & Sriganesh (2008) proposed an innovative approach for real-time fraud detection. A combination of Genetic Algorithm (GA) and Support Vector Machine (SVM), a fraud detection system was proposed by Abdulla, Rakendu & Varghese (2015). GA performed feature selection, while SVM was used for classification.

Frauds related to insurance claims of automobiles are being reported frequently these days. Furlan, Vasilecas & Bajec (2011) proposed a method (tool) for improving the fraud management process in vehicle insurance corporations. Similarly, Artificial Intelligence (AI) has an established impact on machine learning approaches. Topological data analysis could help in financial fraud detection by using case-based reasoning. Where a data bank is populated with well-known financial practices. A solution to the problem of an imbalanced dataset was proposed in Zareapoor & Yang (2017). This approach was tested on the real-time data provided by FICO.

The authors of Li, Sun & Contractor (2017) recommended a graph-mining hybrid approach based on reputation score for fraud detection. Since reputation score is not always available, it could be calculated by careful modeling of edge potential and parameter tuning in the Markov Random Field. Social Network Analysis (SNA) can reveal useful information about groups, their activities, and interaction among actors. Researchers are analyzing social networks to detect financial frauds. Zhou et al. (2017) proposed a ProGuard technique to detect malicious accounts and activities. Using SNA, the authors proposed a method for fraud detection (Colladon & Remondi, 2017; Hamid, 2017; Shaikh & Nazir, 2018).

Ontology is the best way to represent knowledge in a dynamic environment. Rajput, Larik & Haider (2014) proposed an ontology-based system for fraudulent transaction detection. An ontology graph-based system was proposed by Ramaki, Asgari & Atani (2012) for credit card fraud detection. DelMarRoldan-Garci, Garcia-Nieto & Aldana-Montes (2017) proposed an ontology-driven approach for examining and finding inconsistencies, mistakes, and contradictions in Semantic Web Rule Language (SWRL) for fraud prevention.

Numerous fraud detection techniques have been used by financial institutions. Researchers have also proposed different approaches for suspicious transaction detection. Methods like supervised and unsupervised machine learning have been used for the said purpose. Sánchez, Cerda & Serrano (2009) proposed the Association Rule (AR) based methodology for CC fraud detection. The authors applied the proposed solution to the data of retail companies in Chile. A hybrid method using AR and process mining was proposed in Sarno et al. (2015). The authors aimed to solve the problem of fast fraud detection by using the itemset of AR learning. Approaches based on KNN, and outlier detection have been analyzed and implemented by Malini & Pushpa (2017) to optimize solutions for CC fraud detection. Mishra, Gupta & Singh (2017) presented a performance analysis of various approaches used for CC fraud detection. The authors also proposed an Artificial Neural Networks (ANN) model for CC fraud detection.

A classification model was developed in Sahin & Duman (2011) using ANN and logistic regression to solve the problem of CC fraud detection. The model was tested on the real dataset. Xuan et al. (2018) proposed Random Forest (RF) learning method for fraud detection. Two kinds of RF were used to train the pattern of suspicious and non-suspicious transactions. Experiments were conducted using data of e-commerce in China. Halvaiee & Akbari (2014) proposed a nature-inspired based, Artificial Immune System (AIS) technique for suspicious credit card detection. The system proposed had better accuracy and low system cost and response time.

Considering prior research, we propose an improved, feature-rich, and comprehensive ontology-based solution for deterrence and detection of financial fraud. We have created Jena rules for detecting suspicious transactions. Our work also proposes an intimation rule-based alert generation algorithm for generating alerts. We have also presented a comparison of the results of our work with other ontology and non-ontology-based methods.

## Types of Fraud

A variety of financial frauds are being committed nowadays. The most common ones are bank frauds, corporate frauds, and insurance frauds (West & Bhattacharya, 2016). Our focus in this research is on bank frauds. Bank frauds could be of many types. A brief description of common bank frauds is listed below.

### Credit card fraud

CC fraud is the unauthorized use of a CC to perform illegal transactions. CC frauds are often committed by using stolen credit or debit cards. The development of an accurate system for CC fraud detection is a critical problem. Many fraud detection techniques have been proposed by researchers for CC fraud detection. Behera & Panigrahi (2015) proposed a three-layered system for CC fraud detection using fuzzy clustering and neural network. In the first phase, the system performs verification of card details. It then calculates suspicious scores by using fuzzy clustering. Finally, the solution performs suspicious activity detection.

### Money laundering

The process of hiding the source of illegitimate money is known as Money Laundering (ML). ML fraud is performed by transferring money via shell corporations, bank accounts, etc. The key reason behind any fraud is to get illegal financial benefits. Detecting ML is a challenge since fraudsters often find new ways to launder money. A lot of ML detection systems and techniques have been analyzed and practiced in recent years. A hybrid of Hash-Based Association (HBA) and Graph-Theoretic (GT) method was used by Suresh, KT & Sweta (2016) and Pooja et al. (2018) for ML detection, respectively. This method identified the traversal path of the laundered money using the HBA approach. Moreover, it detected the agent of ML by using the GT Approach. Carnaz, Nogueira & Antunes (2017) proposed an ontology-based framework to detect ML.

### Online transaction fraud

**Figure 2:**
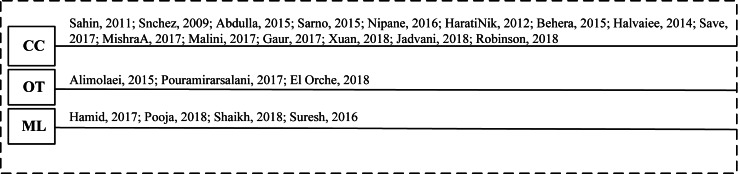
Timeline of financial fraud detection methods.

An online transaction (also known as a PIN-debit transaction) is a process of transferring money or funds online. Online Transaction (OT) fraud is an illegitimate transaction, which occurs via the internet. The payment system has five entities, *i.e.,* cardholders, merchants, card issuers, acquirers, and a payment corporation network. These entities are involved in financial transactions (El Orche, Bahaj & Alhayat, 2018). The problem of OT fraud detection continues to grow. An account of ongoing research to detect OT frauds in financial institutes is present in Fig. 2. The figure also presents the timeline of (CC, ML, and OT) fraud detection techniques reviewed in this article.

## System Model and Problem Formulation

In this section, we discuss our proposed system model and the problem formulation.

### System description

**Figure 3:**
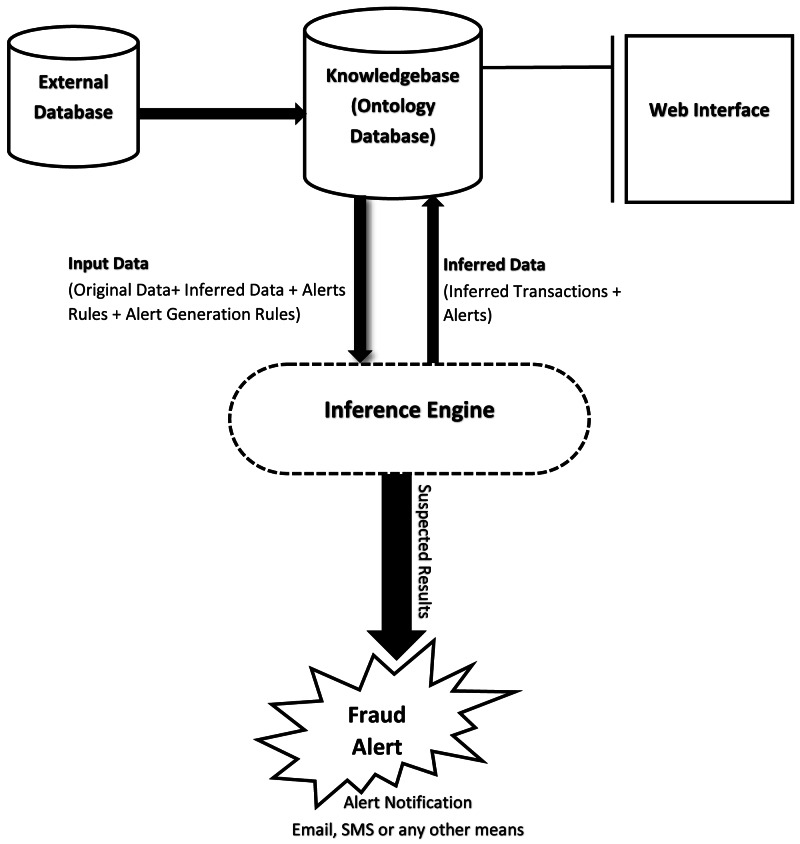
Overview of system functionality.

In this section, we present an enhanced financial fraud detection system. Our ontology-based alert model has added features, *i.e.,* severity levels of alerts based on intimation rules. As a result, our proposed solution performs better. A high-level system architecture is shown in Fig. 3, and the proposed IRB alert generation algorithm is shown in Algorithm 1. Furthermore, the step-by-step execution of the proposed system are described below:

• The first step will be to extract data from external data source(s), *e.g.,* relational database(s). This data will then be preprocessed and saved in the ontological database. After that, each account’s transaction threshold will be calculated. This threshold will then be utilized by the inference engine during rules evaluation against each account’s transaction. The use of dynamic threshold will allow the system to be more effective as it will give a view of the transactional behavior of the customer. Moreover, with the help of a dynamic threshold, the system will adapt to the changing behavior of the customer with time.
• The rules used by the inference engine will define the criteria of setting the severity level of a suspicious alert. If an alert was previously generated for a customer’s account, then, the system might increase the hit count in the alert. The solution will also set the alert-id of the previous alert as the parent-id of the current alert and will increase the severity level. This will allow the system to detect recurring suspicious transactions. Chaining alerts together will also result in a trace of similar alerts which could later be used for inspection & audit.
• As mentioned earlier, the alerts will be generated based on intimation-rules with a certain severity level. The system can generate three different levels of alerts as described below.     Level 1: Suspected alert, when the first occurrence is identified (Severity Level: Low).     Level 2: Investigation Required (Severity Level: Medium).     Level 3: Fraud Detected (Severity Level: High).The severity levels can be used by the fraud notification module to generate emails or SMS etc.

 
_______________________ 
Algorithm 1 IRB Alert Generation Algorithm_________________________________________ 
  1:  Input Data:- Original data, Inferred data, Alert rules, Alert generation rules 
  2:  Output:- Alert notifications, Transaction IRI, Transaction ID, Severity level 
  3:  Data entry in the ontological database 
  4:  Data preprocessing and saving 
  5:  for All data from relational a database to resource description framework 
     store do 
 6:       Calculate account transaction thresholds 
  7:       Apply rules (executed by inference engine) 
  8:       if Indicate risks then 
 9:            Apply intimation rule 
10:            if  Severity level ≥ high  then 
11:                  Pass through severity levels 
12:                  If fraud detected!! 
13:                  Generate alert notifications 
14:            end if 
15:            Return Transaction IRI, ID, Severity level 
16:       end if 
17:  end for____________________________________________________________________________    


### Problem formulation

We formulate the problem of financial fraud detection as a single-objective optimization problem. Suppose, there are two transactions T_cml_ and T_cnr_.

(1) \begin{eqnarray*}{T}_{\mathrm{cml},}=\{ {T}_{\mathrm{cml},1},{T}_{\mathrm{cml},2},{T}_{\mathrm{cml},3},..,{T}_{\mathrm{cml,m}}\} \end{eqnarray*}

(2) \begin{eqnarray*}{T}_{\mathrm{cnr,}}=\{ {T}_{\mathrm{cnr},1},{T}_{\mathrm{cnr},2},{T}_{\mathrm{cnr},3},..,{T}_{\mathrm{cnr,n}}\} \end{eqnarray*}

Where, T_cml_ are transactions from commercial account and T_cnr_ are transactions from consumer account.

(3) \begin{eqnarray*}T={T}_{\mathrm{cml}}\cup {T}_{\mathrm{cnr}}\end{eqnarray*}

In transactions T, fraudulent F and legitimate L transactions are the subset of transaction T, (*F* ⊂ *T*, *L* ⊂ *T*). Whereas, F and L contains the number of fraudulent and legitimate transactions, respectively.

(4) \begin{eqnarray*}F=\{ {F}_{1},{F}_{2},{F}_{3},..,{F}_{m}\} \end{eqnarray*}

(5) \begin{eqnarray*}L=\{ {L}_{1},{L}_{2},{L}_{3},..,{L}_{n}\} \end{eqnarray*}

(6) \begin{eqnarray*}T=F\cup L\end{eqnarray*}

Transaction is either legitimate or fraudulent, as states shown in Eq. (7)

(7) \begin{eqnarray*}{\alpha }_{ij}= \left\{ \begin{array}{@{}ll@{}} \displaystyle 1,\hspace*{10.00002pt}&\displaystyle \text{is fraudulent},\\ \displaystyle 0,\hspace*{10.00002pt}&\displaystyle \text{is legitimate}. \end{array} \right. \end{eqnarray*}

The objective is to minimize fall-out and miss rate as shown in the following equation.

(8) \begin{eqnarray*}Minimize\sum _{i=1}^{n}\sum _{j=1}^{m}F{N}_{ij}+F{P}_{ij}\ast {\alpha }_{ij}\end{eqnarray*}

Where False Negative (FN) is the number of objects of set F, which were expected as an object of L incorrectly. False Positive (FP) is the number of objects of set L, which were expected as an object of F incorrectly. FP is also known as the fall-out rate.

## Ontology Construction Methodology

In this work, METHONTOLOGY (Corcho et al., 2005) is used to illustrate the construction of an ontology. This framework allows ontologies to be modelled using graphical representation. With a graphical representation, a specialist in one domain can perceive the ontology from another domain. METHONTOLOGY has several phases. It also identifies management, support and development activities. Management activities include control, quality assurance, and schedule. Support activities involve configuration management, documentation, evaluation, integration and knowledge acquisition. Development activities include specification, conceptualization, formalization, implementation, and maintenance.

### Formal representation of FFD ontology

An ontology represents knowledge in an easily shareable and reusable manner. It describes the terms and their relationships within the given domain. An ontology consists of concept, relation, and attribute identifiers along with data types (Cimiano, 2006). Moreover, the structure of ontology can be represented as formal logic as shown below:

(9) \begin{eqnarray*}O=Ontology=(C,{\leq }_{t},S,P)\end{eqnarray*}

where C is the set of classes, ≤_*t*_ on C is called concept hierarchy. S stands for subclasses, P represents predicate (relationships). Moreover, they can be represented as follows:

(10) \begin{eqnarray*}C= \left( \right. \prod _{i=1}^{n}{C}_{i},{\leq }_{t} \left( \right. \end{eqnarray*}

where i = (1,2,3, …,n) and ≤ fulfills the conditions as shown below.

(11) \begin{eqnarray*}\forall a,\hspace*{5.69054pt}(a\leq a)\hspace*{5.69054pt}\end{eqnarray*}

(12) \begin{eqnarray*}\forall a\hspace*{2.84526pt}\forall b,\hspace*{2.84526pt}(a\leq b\wedge b\leq a\Longrightarrow a=b)\hspace*{5.69054pt}\end{eqnarray*}

(13) \begin{eqnarray*}\forall a\hspace*{2.84526pt}\forall b\hspace*{2.84526pt}\forall c,\hspace*{5.69054pt}(a\leq b\wedge b\leq c\Longrightarrow a\leq c)\hspace*{2.84526pt}\end{eqnarray*}

(14) \begin{eqnarray*}\forall a\hspace*{11.38109pt}(a\leq top\hspace*{2.84526pt}element)\hspace*{8.53581pt}\end{eqnarray*}

## FFD Ontology Implementation

In this section, we introduce our ontology based FFD Model and its rules for detecting suspicious transactions. Our system is made of three main components:

• Ontology Development
• Ontology Reasoning
• Results by Querying on Inferred Ontology

### Ontology development

The first step of ontology development is to perform data preprocessing. The data items from this step are selected and transformed into an ontology. All the irrelevant and redundant information is filtered out to make the data more meaningful. This process of filtering out data is often referred to as dataset normalization. Our proposed system models, domain knowledge into ontology and defines rules on top to support reasoning. The inference engine uses these rules to infer new knowledge to aid in the identification of suspicious transactions. The knowledge base used by our proposed system consists of customer transaction data. The ontology model consists of classes, subclasses, objects, datatype properties, and instances. The transactional data contains the amount and their frequency in each interval. We have designed a three-layered ontology as described below.

**i. Conceptualization of the Domain Layer:**

In the domain layer customer’s transactions are modeled in various forms. Classes, subclasses, properties (object/datatype), and instances are created in this layer. The key classes of our proposed ontology are account, person, purposes, suspicious alerts, and transaction types.

**ii. Ontology Layer:**

**Figure 4:**
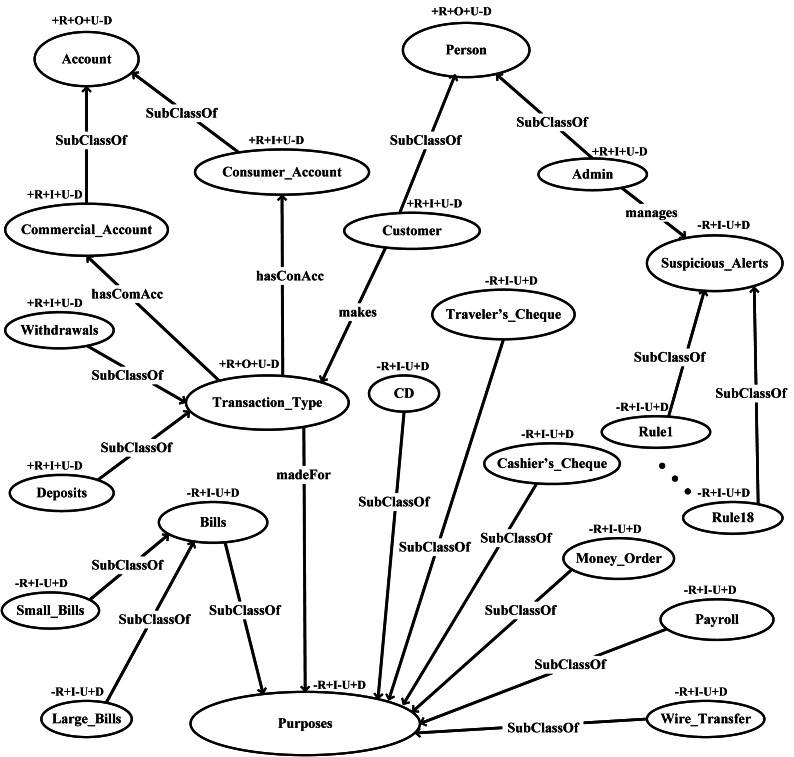
FFD ontology validation through OntoClean.

This layer defines restriction on classes via Ontology Web Language (OWL) to facilitate logic. A graphical representation of the proposed FFD ontology with its classes and subclasses is shown in Fig. 4.

**iii. Rule Layer:** To infer new knowledge from the existing knowledge, rules are developed on top of the ontology OWL layer. In this study, the rules are created in Jena. Jena is a semantic web toolkit (Carroll et al., 2004). It is a Java framework for the creation of applications for the Semantic Web. Three levels of rules are executed by the inference engine. We have created rules based on the Anti Money Laundering (AML) guidelines shared by the financial regulatory authority. The values of the Threshold Amount (TA) can vary depending on the financial institution. The threshold values also depend on the AML guidelines of different countries. For the purposes of this work, we have suggested a few threshold values to aid our proof of concept. Our suggested four TA values are: TA1 is equal to 10000 USD, TA2 is 8000 USD, TA3 is 5000 USD and TA4 is equal to 3000 USD.

### Ontology reasoning

Once the knowledge base is developed, it is populated with transaction records and appropriate rules. The reasoner then infers logical information from the set of asserted facts. The inference rules are commonly specified through an ontology language. Traditional reasoning engines (Pellet, HermiT, FaCT++, etc.) can be used for reasoning (Khamparia & Pandey, 2017). We have used the FaCT++ 1.6.5 reasoning engine in this work.

### Results by querying on inferred ontology

Once the inference engine infers knowledge based on the given rules, the information (asserted or inferred) can be queried. SPARQL is a query language that is often used to get the required information (Sirin & Parsia, 2007). In our work, we have also used SPARQL to query the FFD ontology.

## Ontology Validation

In this section, we discuss our proposed methodology in detail. We also discuss constraints and our assumptions for FFD ontology’s validation.

### OntoClean methodology

In this work, we have used OntoClean (Guarino & Welty, 2004) for ontology verification. It is a formal method for evaluating the ontological sufficiency of taxonomic relationships. The property of a property is known as meta-property. Unity, identity, rigidity, dependency, and essence are meta properties (formal notions) of OntoClean. Meta property can be further classified into three main labels (+, -, ∼). The description of each label is shown in Table 1.

**Table 1:** Description of Meta Properties.

**Meta property**	**Description**
**+R** (Rigid)	All object must be objects of this concept in every possible world.
**-R** (Non-Rigid)	Objects will stop being objects of the concept.
**∼R** (Anti-Rigid)	objects will not any longer be the object of that concept.
**+I** (Identity)	Objects carry unique identification criteria from any parent class.
**-I** (Non-Identity)	There are no identification criteria.
**+O** (Supply Identity)	Objects themselves provide a unique identification criteria.
**+U** (Unity)	Objects are “whole” and have a single unit criteria.
**-U** (Non-Unity)	Objects are “whole” and do not have a single unit criteria.
**∼U** (Anti-Unity)	Objects are not “whole”.
**+D** (Dependence)	Dependency exists.
**-D** (Non-Dependence)	No dependency.

OntoClean has devised a method to characterize properties and classes and their relations in an ontology. OntoClean attaches the meta properties to each concept and removes false relationships. It further checks the consistency, conciseness, and completeness of ontology. In this work, we have used the OntoClean method for the validation of FFD ontology. The proposed ontology is validated by using meta-properties *i.e.,* unity, identity, rigidity, dependency as depicted in Fig. 4. The validation criteria of the OntoClean method are shown below.

### Constraints and assumptions

For validating and ensuring the accuracy of ontology, conditions are applied to classes and properties (Guarino, 2004). Assume, there are two properties, X and Y, when Y subsumes X, so their resulting restrictions hold as follows:

1) If Y has anti-rigid **(∼R)**, then X must have anti-rigid **(∼R)**.

2) An **∼R** property cannot subsume a **+R** property.

3) If Y is rigid **(+R)**, then X must be rigid **(+R)**.

4) An **+R** property cannot subsume a **∼R** property.

5) If Y has identity **(+I)**, then X must have identity **(+I)**.

6) If Y is unity **(+U)**, then X must be unity **(+U)**.

7) If Y is anti-unity **(∼U)**, then X must be anti-unity **(∼U)**.

8) An **∼U** property cannot subsume a **+U** property.

9) If Y has dependence, then X must have dependence **(+D)**.

## Simulation Setup and Results

In this section, we discuss the dataset and simulation tools. We also discuss the evaluation measures and performance comparison of the proposed system.

### Dataset

For experiments, we have used a real dataset. The dataset contained 1048576 individual transactions. In the dataset, transactions are classified on the basis of days, weeks, and months. The key values from our dataset are the total deposit and withdrawal amount. The frequency of deposits and withdrawals based on days, weeks, and months is also present in the dataset. The transaction records are separated by deposits and withdrawal to capture the flow of money.

### Simulation tools

The experiments were conducted on a Haier Laptop 7G-5 h with 1.70 GHz Intel Core i3 and 4Gb RAM running Windows 10. In this work, we have used simple tools for compiling results. The tools used are listed below:

1. Eclipse IDE 2018-09 (4.9.0)
2. Java 1.8.0_151
3. Protege 5.2.0 Ontology Editor
4. SPARQL query language
5. Apache Jena 3.9.0 Semantic Web Framework
6. FaCT++ 1.6.5 Reasoner

For writing and compiling code, we have used the Eclipse IDE. We used Java for writing our logic along with a Java-based Apache framework: Jena. With Jena, we manipulated ontologies and rules whilst FaCT++ 1.6.5 was used to infer knowledge from the knowledge base. We used Protege 5.2.0 to develop FFD ontology and SPARQL query language to query the financial fraud detection ontology. In the next subsection, we discuss the experimental results of FFD in detail.

### Evaluation measures

Before we describe the experimental results, we first introduce the metrics. In this work, the metrics we used for performance comparison of the FFD system are accuracy, precision, recall, F-measure, and Matthews Correlation Coefficient (MCC). Furthermore, the formulas of the aforementioned measures are presented below:

(15) \begin{eqnarray*}Accuracy= \frac{TP+TN}{TP+FP+TN+FN} \end{eqnarray*}

(16) \begin{eqnarray*}Precision= \frac{TP}{(TP+FP)} \end{eqnarray*}

(17) \begin{eqnarray*}Recall= \frac{TP}{(TP+FN)} \end{eqnarray*}

(18) \begin{eqnarray*}F-measure= \frac{2\ast Precision\ast Recall}{Precision+Recall} \end{eqnarray*}

(19) \begin{eqnarray*}MCC= \frac{TP\ast TN-FP\ast FN}{\sqrt{(TP+FP)(TP+FN)(TN+FP)(TN+FN)}} \end{eqnarray*}

Where,

• True Positive (TP) = Number of Legitimate Transactions (LTs) which were identified correctly.
• False Negative (FN) = Number of LTs which were expected as Fraudulent Transactions (FTs) incorrectly.
• True Negative (TN) = Number of FTs which were identified correctly.
• False Positive (FP) = Number of FTs which were expected as LTs incorrectly.

### Results and performance comparison

**Figure 5:**
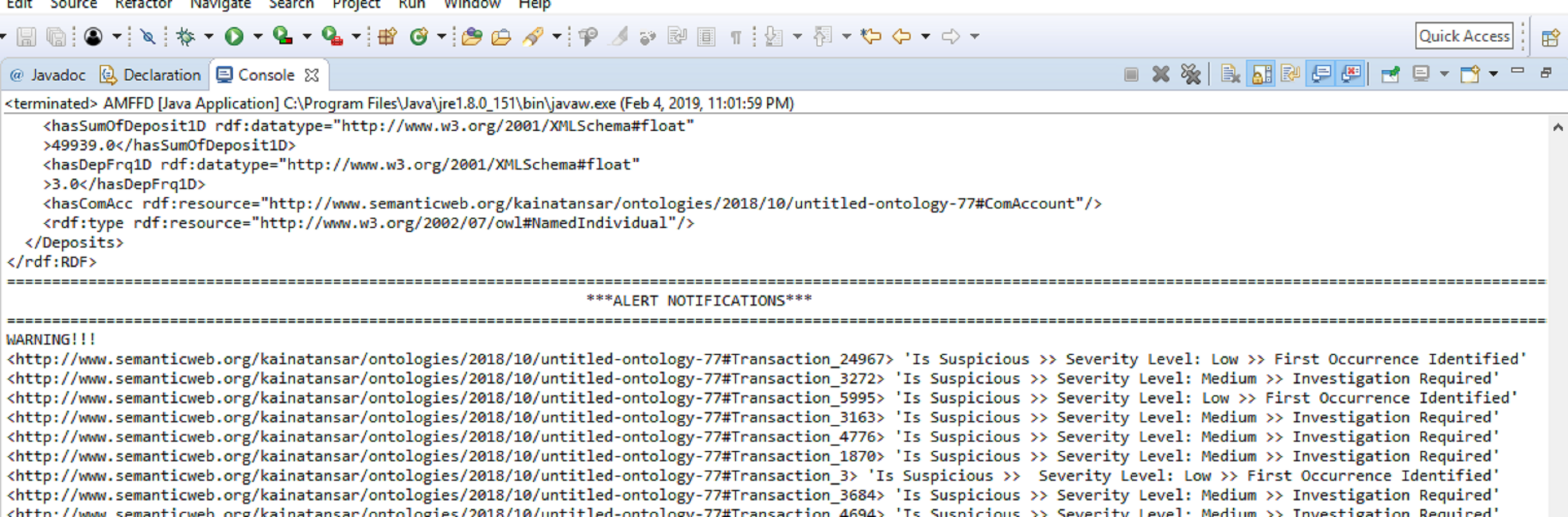
Alerts generated by FFD system.

Our proposed solution generates alerts at the onset of suspicious activity. Alerts can be generated with either of the three severity levels discussed in ‘System Model and Problem Formulation’. Alert notifications generated by the FFD system are shown in Fig. 5. We have compared our proposed solution with ontological and non-ontological solutions. For ontological solutions, our comparison is based on the number of classes, subclasses, and properties *i.e.,* data and object. The ontological solutions in comparison have a small number of classes and properties. This means that a narrower domain was considered to solve the issue of financial fraud. Since our solution covers a wider area of the financial fraud domain with a greater number of classes and properties. We believe that our solution is better at detecting and deterring the threat of financial fraud. Table 2 shows a comparative analysis of FFD and other ontologies on the basis of classes, subclasses, and properties.

**Table 2:** Comparison with Ontology-Based Systems.

**Reference**	**Classes + SubClasses**	**Properties**
El Orche & Bahaj (2020)	9	7
Sahri, Shuhidan & Sanusi (2018)	9	2
Rajput, Larik & Haider (2014)	19	10
Attigeri et al. (2018)	8	2
El Orche, Bahaj & Alhayat (2018)	5	6
Our proposed FFD	40	22

We have also compared our solution with other non-ontological solutions on the basis of various benchmarks.

**Figure 6:**
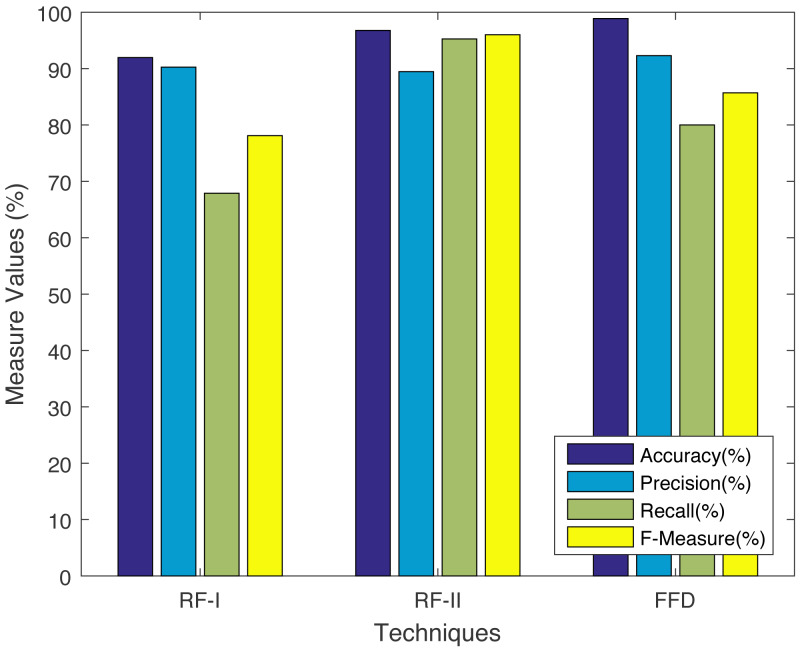
Comparison with non-ontology based techniques.

We are using metrics, *i.e.,* accuracy, precision, recall, and F-measure. The said comparison between FFD and non-ontological solutions *e.g.,* RF-I and RF-II, (Xuan, 2018) are shown in Fig. 6. Before we do the comparison, we need to calculate the accuracy, precision, recall, and F-measure using Eqs. (15)–(18). The results show that the accuracy and precision of the FFD system increases, while the F-measure decreases when compared to RF-II. The recall achieves the greatest value when compared to RF-I. It is evident from Fig. 6 that our solution achieves the highest precision and accuracy among all benchmarks.

## Conclusions And Future Work

This article introduces fraud trends in financial institutions. We describe data representational models and the advantages of using ontologies over databases. Later, we propose an enhanced ontology-based FFD system for fraud detection and deterrence. Our work also presents an IRB alert generation algorithm for alert generation. We have also developed a taxonomy of literature review. The strength of our ontology-based alert model is its ability to reason. Reasoning capability in ontologies makes it possible to derive inexplicit facts. Our proposed solution generates alerts with appropriate severity levels. It also excludes dead alerts which makes our solution reliable, quicker, and efficient. In the future, we aim to investigate the efficacy of the FFD system in other fraud-prone domains. We believe that domains, *i.e.,* telecommunication, internet marketing, and insurance fraud are also a good place to test our solution.

## Supplemental Information

10.7717/peerj-cs.649/supp-1 Supplemental Information 1 Jena Rules (.txt file)

10.7717/peerj-cs.649/supp-2 Supplemental Information 2 Ontology Source code (.txt file)

10.7717/peerj-cs.649/supp-3 Supplemental Information 3 A set of main rule model

10.7717/peerj-cs.649/supp-4 Supplemental Information 4 A set of main rules with corresponding Jena syntax

## Additional Information and Declarations

### Data Availability

The following information was supplied regarding data availability: The JENA rules, ontology code, and main rules are available in the Supplemental Files.
