# Supplementary figures and images for: A semantic rule based digital fraud detection
Source: PeerJ Comput Sci. 2021 Aug 3;7:e649. doi: 10.7717/peerj-cs.649 (PMC8356649; doi:10.7717/peerj-cs.649)

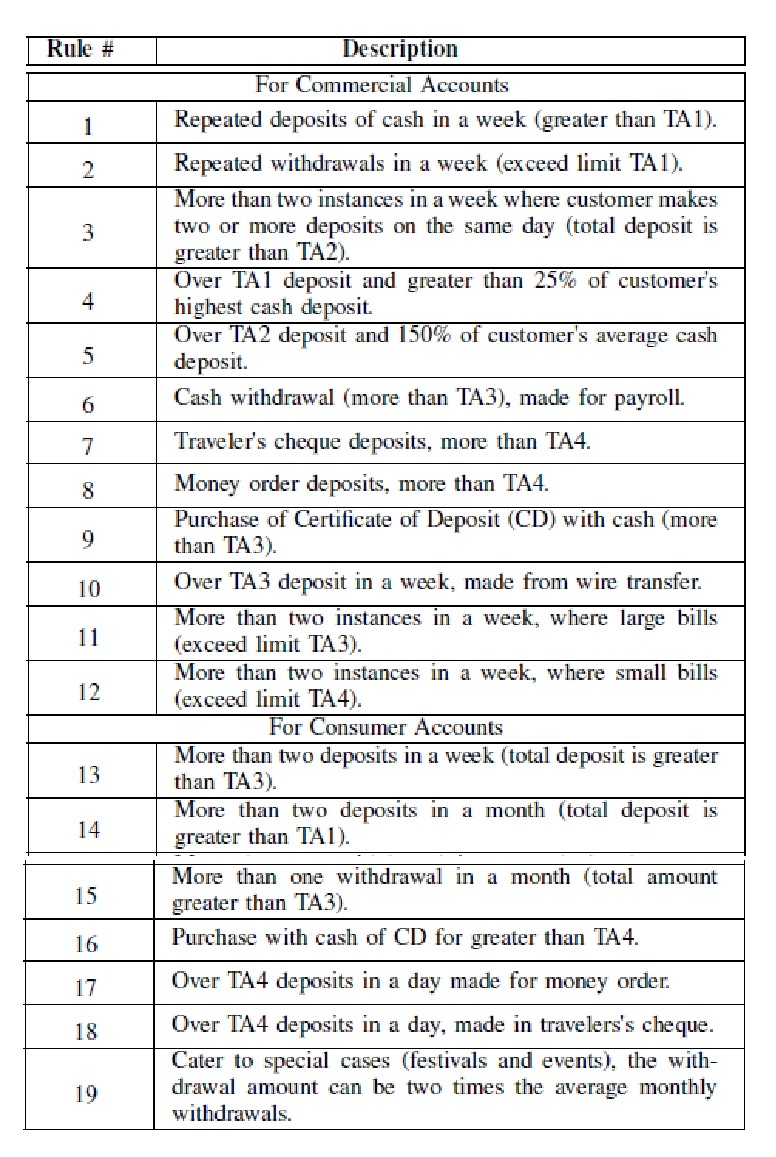

Supplement: Supplemental Information 3 [file peerj-cs-07-649-s003.jpg]
