# Supplementary material for: A semantic rule based digital fraud detection
Source: PeerJ Comput Sci. 2021 Aug 3;7:e649. doi: 10.7717/peerj-cs.649 (PMC8356649; doi:10.7717/peerj-cs.649)
Supplement: Supplemental Information 4 [file peerj-cs-07-649-s004.pdf]

Jena Rules for Generating Alert Notifications

Rule #	Description
1	[Rulee1:(?AC rdf:type dc:Commercial_Account)(?DT rdf:type dc:Deposits)(?DT dc:hasComAcc ?AC)(?DT dc:hasSumOfDeposit7Ds ?d7AMT)(?DT dc:hasDepFrq7Ds ?d7FRQ) greaterThan(?d7AMT ,10000) greaterThan(?d7FRQ ,2) − > print(?DT,'Is Suspicious >> Severity Level: Medium >> Investigation Required')]
2	[Rulee2: (?AC rdf:type dc:Commercial_Account) (?WT rdf:type dc:Withdrawals) (?WT dc:hasComAcc ?AC) (?WT dc:hasSumOfWithdrawal7Ds ?w7AMT) (?WT dc:hasWithdrawalFrq7Ds ?w7FRQ) greaterThan(?w7AMT ,10000) greaterThan(?w7FRQ ,2) − > print(?WT,'Is Suspicious >> Severity Level: High >> Fraud Detected')]
3	[Rulee3: (?AC rdf:type dc:Commercial_Account) (?DT rdf:type dc:Deposits) (?DT dc:hasComAcc ?AC) (?DT dc:hasSumOfDeposit1D ?d1AMT)(?DT dc:hasDepFrq1D ?d1FRQ) (?DT dc:hasDepFrq7Ds ?d7FRQ) greaterThan(?d1AMT ,8000) greaterThan(?d1FRQ ,2) greaterThan(?d7FRQ ,3) − > print(?DT,'Is Suspicious >> Severity Level: Low >> First Occurrence Identified')]
4	[Rulee4: (?AC rdf:type dc:Commercial_Account) (?DT rdf:type dc:Deposits) (?DT dc:hasComAcc ?AC) (?DT dc:hasMaxDep ?mxAMT) greaterThan(?mxAMT ,10000) − > print(?DT,'Is Suspicious >> Severity Level: Medium >> Investigation Required')]
5	[Rulee5: (?AC rdf:type dc:Commercial_Account) (?DT rdf:type dc:Deposits) (?DT dc:hasComAcc ?AC) (?DT dc:hasAvgDep ?avgAMT) greaterThan(?avgAMT ,10000) − > print(?DT,'Is Suspicious >> Severity Level: High >> Fraud Detected')]
6	[Rulee6: (?AC rdf:type dc:Commercial_Account) (?WT rdf:type dc:Withdrawals) (?WT dc:hasComAcc ?AC) (?WT dc:hasSumOfWithdrawal1D ?w1AMT) (?WT dc:hasPurpose ?PP) equal(?PP,"Payroll") greaterThan(?w1AMT ,5000) − > print(?WT,'Is Suspicious >> Severity Level: Low >> First Occurrence Identified')]
7	[Rulee7: (?AC rdf:type dc:Commercial_Account) (?DT rdf:type dc:Deposits) (?DT dc:hasComAcc ?AC) (?DT dc:hasSumOfDeposit1D ?d1AMT) (?DT dc:hasPurpose ?PP) equal(?PP,"Money_Order") greaterThan(?d1AMT ,3000) − > print(?DT,'Is Suspicious >> Severity Level: High >> Fraud Detected')]
8	[Rulee8: (?AC rdf:type dc:Commercial_Account) (?DT rdf:type dc:Deposits) (?DT dc:hasComAcc ?AC) (?DT dc:hasSumOfDeposit1D ?d1AMT) (?DT dc:hasPurpose ?PP) equal(?PP,"Traveler's_Cheque") greaterThan(?d1AMT ,3000) − > print(?DT,'Is Suspicious >> Severity Level: High >> Fraud Detected')]
9	[Rulee9: (?AC rdf:type dc:Commercial_Account) (?DT rdf:type dc:Deposits) (?DT dc:hasComAcc ?AC)(?DT dc:hasSumOfDeposit1D ?d1AMT) (?DT dc:hasPurpose ?PP) equal(?PP,"CD") greaterThan(?d1AMT ,5000)− > print(?DT,'Is Suspicious >> Severity Level: Medium >> Investigation Required')]
10	[Rulee10: (?AC rdf:type dc:Commercial_Account) (?DT rdf:type dc:Deposits) (?DT dc:hasComAcc ?AC) (?DT dc:hasSumOfDeposit7Ds ?d7AMT) (?DT dc:hasPurpose ?PP) equal(?PP,"Wire_Transfer") greaterThan(?d7AMT ,5000) − > print(?DT,'Is Suspicious >> Severity Level: Medium >> Investigation Required')]
11	[Rulee11: (?AC rdf:type dc:Commercial_Account) (?DT rdf:type dc:Deposits) (?DT dc:hasComAcc ?AC) (?DT dc:hasSumOfDeposit7Ds ?d7AMT)(?DT dc:hasDepFrq7Ds ?d7FRQ) (?DT dc:hasPurpose ?PP) equal(?PP,"Small_Bills") greaterThan(?d7AMT ,3000) greaterThan(?d7FRQ ,2) − > print(?DT,'Is Suspicious >> Severity Level: Medium >> Investigation Required')]
12	[Rulee12: (?AC rdf:type dc:Commercial_Account) (?DT rdf:type dc:Deposits) (?DT dc:hasComAcc ?AC) (?DT dc:hasSumOfDeposit7Ds ?d7AMT)(?DT dc:hasDepFrq7Ds ?d7FRQ) (?DT dc:hasPurpose ?PP) equal(?PP,"Large_Bills") greaterThan(?d7AMT ,5000) greaterThan(?d7FRQ ,2) − > print(?DT,'Is Suspicious >> Severity Level: Medium >> Investigation Required')]
13	[Rulee13: (?ACC rdf:type dc:Consumer_Account) (?DT rdf:type dc:Deposits) (?DT dc:hasConAcc ?ACC) (?DT dc:hasSumOfDeposit7Ds ?d7AMT) (?DT dc:hasDepFrq7Ds ?d7FRQ) greaterThan(?d7AMT ,5000) greaterThan(?d7FRQ ,2) − > print(?DT,'Is Suspicious >> Severity Level: Low >> First Occurrence Identified')]
14	[Rulee14: (?ACC rdf:type dc:Consumer_Account) (?DT rdf:type dc:Deposits) (?DT dc:hasConAcc ?ACC) (?DT dc:hasSumOfDeposit30Ds ?d30AMT) (?DT dc:hasDepFrq30Ds ?d30FRQ) greaterThan(?d30AMT ,10000) greaterThan(?d30FRQ ,2) − > print(?DT,'Is Suspicious >> Severity Level: Low >> First Occurrence Identified')]
15	[Rulee15: (?ACC rdf:type dc:Consumer_Account) (?DT rdf:type dc:Deposits) (?DT dc:hasConAcc ?ACC) (?DT dc:hasSumOfDeposit1D ?d1AMT) (?DT dc:hasPurpose ?PP) equal(?PP,"CD") greaterThan(?d1AMT ,3000) − > print(?DT,'Is Suspicious >> Severity Level: Low >> First Occurrence Identified')]
16	[Rulee16: (?ACC rdf:type dc:Consumer_Account) (?WT rdf:type dc:Withdrawals) (?WT dc:hasConAcc ?ACC) (?WT dc:hasSumOfWithdrawal30Ds ?w30AMT) (?WT dc:hasWithdrawalFrq30Ds ?w30FRQ) greaterThan(?w30AMT ,5000) greaterThan(?w30FRQ ,2) − > print(?WT,'Is Suspicious >> Severity Level: Low >> First Occurrence Identified')]
17	[Rulee17: (?ACC rdf:type dc:Consumer_Account) (?DT rdf:type dc:Deposits) (?DT dc:hasConAcc ?ACC) (?DT dc:hasSumOfDeposit1D ?d1AMT) (?DT dc:hasPurpose ?PP) equal(?PP,"Money_Order") greaterThan(?d1AMT ,3000) − > print(?DT,'Is Suspicious >> Severity Level: Low >> First Occurrence Identified')]
18	[Rulee18: (?ACC rdf:type dc:Consumer_Account) (?DT rdf:type dc:Deposits) (?DT dc:hasConAcc ?ACC) (?DT dc:hasSumOfDeposit1D ?d1AMT) (?DT dc:hasPurpose ?PP) equal(?PP,"Traveler's_Cheque") greaterThan(?d1AMT ,3000) − > print(?DT,'Is Suspicious >> Severity Level: Low >> First Occurrence Identified')]
19	[Rulee19: (?ACC rdf:type dc:Consumer_Account) (?WT rdf:type dc:Withdrawals) (?WT dc:hasConAcc ?ACC) (?WT dc:hasSumOfWithdrawal30Ds ?w30AMT) (?WT dc:hasAvgDep ?avgWithdrawal) product(?avgWithdrawal, ?2, ?2XavgWithdrawal) greaterThan(?w30AMT, ?2XavgWithdrawal) − > print(?WT,'Is Unsuspicious >> Severity Level: Down >> For special event E its severity level will be lower as it is expected to have higher withdrawals on this event ')]

It should be mentioned that the values of Threshold Amount (TA) are not fixed. These threshold values may vary depending on the AML guidelines used in different countries. However, in this work, we have suggested these values as TA1 is equal to 10000 USD, TA2 is 8000 USD, TA3 is 5000 USD and TA4 is equal to 3000 USD.
